# Complete Genome Sequences of Three Strains of Coxsackievirus A7

**DOI:** 10.1128/genomeA.00146-13

**Published:** 2013-04-11

**Authors:** Jani Ylä-Pelto, Satu Koskinen, Eveliina Karelehto, Eleonora Sittig, Merja Roivainen, Timo Hyypiä, Petri Susi

**Affiliations:** Department of Virology, University of Turku, Turku, Finlanda;; Degree Program of Biotechnology and Food Technology, Turku University of Applied Sciences, Turku, Finlandb;; National Institute for Health and Welfare, Helsinki, Finlandc

## Abstract

Genomes of three strains (Parker, USSR, and 275/58) of coxsackievirus A7 (CV-A7) were amplified by the long reverse transcription (RT)-PCR method and sequenced. While the sequences of Parker and USSR were identical, the similarities of 275/58 to the CV-A7 reference sequence, accession no. AY421765, were 82.6% and 96.2% for nucleotides and amino acids, respectively.

## GENOME ANNOUNCEMENT

Coxsackievirus A7 (CV-A7) belongs to the *Enterovirus A* species (within the genus *Enterovirus*, family *Picornaviridae*), which is phylogenetically distinct from other enterovirus species (1, 2). CV-A7 was widely detected in the 1950s and 1960s during paralytic epidemics but has not been detected recently (3–5). It is, together with poliovirus and enterovirus 71, one of the few picornaviruses that has been associated with outbreaks of flaccid paralysis (6–11). This report describes the sequencing of the genomes of three CV-A7 strains, including a strain known as polio-4 (12).

Three CV-A7 strains, Parker (13), USSR (12), and 275/58 (14), were originally obtained from the American Type Culture Collection (VR-166, VR-319, and VR-673 for Parker, USSR, and 275/58, respectively) and propagated and purified as described before (15). Purified strains were serotyped by neutralizing CV-A7-specific World Health Organization (WHO) horse antiserum and typed by partial sequencing of VP1 prior to amplification by long reverse transcription (RT)-PCR and full-length sequencing (16). Integrity of the sequences was verified by tagging of the 5′-terminal PCR primers with the T7 promoter region for virus rescue after transfection of BSR-T7/5 cells (17), followed by visualization of the virus by immunofluorescence using rabbit anti-CV-A7 antiserum. Raw sequence data were assembled using BioEdit v. 7.0.9.0. Nucleic acid and protein sequence alignments were done with ClustalW2 program v. 2.0.12 with default settings (18). Differences between viruses were calculated with the PHYLIP software package (phylogeny inference package, v. 3.68) dnadist and Protdist programs (19). Bootscan analysis was performed using the SimPlot program (version 3.5.1).

At present, there is only a single CV-A7 reference sequence in GenBank (for Parker, cited here with GenBank accession no. AY421765) (1). While the VP1 sequences of Parker and USSR were 100% similar to each other and to that of accession no. AY421765, the VP1 of 275/58 was only 83.3% similar at the nucleotide level. However, the similarity at the amino acid level was 95.1%, which together with the antibody neutralization makes 275/58 a strain of the CV-A7 (sero)type. The full genome of the Parker strain was resequenced because of the similarity between the VP1 sequences of accession no. AY421765, Parker, and USSR. The overall genome organizations of the CV-A7 strains were similar to those of other enteroviruses. Parker, USSR, and 275/58 were 7,403 bp, 7,404 bp, and 7,405 bp in length, respectively. A large open reading frame (6,579 bp) encodes a polyprotein precursor of 2,193 amino acids. Capsid (P1) gene sequences of 275/58 were 81.6 to 84.4% identical to those of accession no. AY421765 (with 94.2 to 98.3% amino acid identity). P2 gene sequences were 80% identical (with 94.2 to 98.8% amino acid identity) and P3 genes were 75.8 to 81.5% identical (with 94 to 98.8% amino acid identity) to those of accession no. AY421765. Comparison with other enteroviruses confirmed that 275/58 was most closely related to members within EV-A. Genomes contain 5′ untranslated regions (UTRs) (742 nucleotides [nt], 742 nt, and 743 nt, respectively) and 3′ UTRs (82 nt, 83 nt, and 83 nt, respectively). The three sequences have similar G+C contents for the complete genome (47.02%, 46.93%, and 47.81%, respectively). Bootscan analysis revealed no recombination events between CV-A7 and EV-A types. Sequence differences between Parker/USSR and 275/58 were scattered throughout the genome.

### Nucleotide sequence accession numbers. 

The sequences have the following GenBank accession numbers: GU942823 (Parker), GU942822 (USSR), and GU9428204 (275/58).
